# Quadricuspid Aortic Valve: A Comprehensive Review

**DOI:** 10.5935/1678-9741.20160090

**Published:** 2016

**Authors:** Shi-Min Yuan

**Affiliations:** 1 The First Hospital of Putian, Teaching Hospital, Fujian Medical University, Putian, Fujian Province, China.

**Keywords:** Aortic Valve Insufficiency, Cardiac Surgical Procedures, Heart Valve Diseases

## Abstract

Quadricuspid aortic valve (QAV) is a rare congenital heart disease. The functional status of QAV is predominantly a pure aortic regurgitation. Clinical manifestations of patients with a QAV depend on the functional status of the QAV and the associated disorders. Significant valvular regurgitation and (or) stenosis is often present with subsequent operation performed at the fifth to sixth decade of life. The functional status of QAV is predominantly regurgitant; whereas pure stenotic QAV can be as few as in only 0.7% of the patients. QAV is usually an isolated anomaly, but other congenital heart defects can be present in 18-32% of the patients. About one-fifth of them require a surgical operation. Tricuspidalization is a preferred technique for QAV repair. As not all the patients with a QAV necessarily warrant a surgical operation, decision-making in patient selection and surgical procedure of choice are crucial. Antibiotic prophylaxis against infective endocarditis is necessary in the QAV patients with unequal-sized cusps.

**Table:** 

**Abbreviations, acronyms & symbols**	
AR	=Aortic regurgitation
QAV	=Quadricuspid aortic valve

## INTRODUCTION

Quadricuspid aortic valve (QAV) is a rare congenital heart disease with an incidence of 0.00028-0.00033% in autopsy series^[^^1^^]^, 0.0059-0.0065% for patients undergoing transthoracic echocardiographic examinations^[^^2^^]^ and 0.05-1% for those receiving aortic valve replacements for aortic regurgitation (AR)^[^^3^^,^^4^^]^. With the advent of echocardiography and other imaging diagnostic techniques, QAVs are increasingly reported^[^^5^^]^.

Debates remain in the management strategies of the patients with a QAV in terms of surgical indication, surgical procedure of choice and antibiotic prophylaxis against infective endocarditis.

The aim of the present study is to describe the clinical features and treatment strategies of QAV.

## MECHANISMS

The mechanisms of QAV development remain unclear. It was believed to be anomalous septation of the conotruncus and abnormal septation of one of the endocardial cushions as a result of an inflammatory episode^[^^6^^]^. Aberrant cusp formation may represent abnormal fusion of the aorticopulmonary septum or abnormal mesenchymal proliferation in the truncus arteriosus^[^^7^^]^.

## CLASSIFICATIONS

**Fig. 1:**
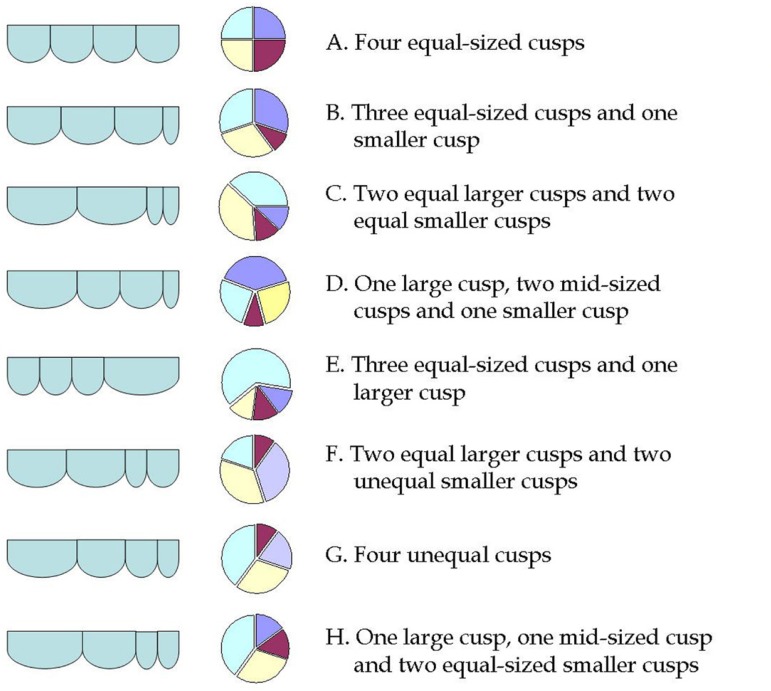
Hurwitz & Roberts^[^^8^^]^ classification of quadricuspid aortic valve with Vali et al.^[^^9^^]^ supplement.

**Fig. 2:**
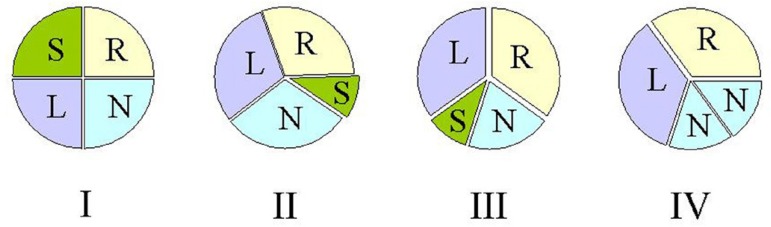
Nakamura et al.^[^^11^^]^ simplified classification of quadricuspid aortic valve. L=left coronary cusp; N=non-coronary cusp; R=right coronary cusp; S=supernumerary cusp

There are two classification schemes. The Hurwitz & Roberts^[^^8^^]^ classification, based on the relative size of the supranumerary cusp, divides QAVs into 7 types from A to G, to which Vali et al.^9^^]^ supplemented with a type H (Figure 1). Types A, B and C represent more than 85% of the cases; while type D variant is very rare^[^^10^^]^. Nakamura et al.^[^^11^^]^ designed a simplified classification by focusing on the position of the supernumerary cusp: type I, supernumerary cusp between the left and right coronary cusps; type II, supernumerary cusp between the right and non-coronary cusps; type III, supernumerary cusp between the left and noncoronary cusps; and type IV, unidentified supernumerary cusp as of two equal-sized smaller cusps (Figure 2). Types I and II of the simplified classification are the same as types A and B of Hurwitz & Roberts^[^^8^^]^. Nakamura et al.^[^^11^^]^ reviewed 42 patients with a QAV, and disclosed that the four types accounted for 23.8%, 30.9%, 7.1% and 4.9%, respectively. They also found the location of the supernumerary cusp did not influence the clinical outcomes^[^^11^^]^. Pirundini et al.^[^^4^^]^ found type II QAV account for 39%.

## AORTIC VALVE FUNCTION

The functional status of QAV is predominantly a pure AR^[^^4^^,^^12^^]^, *i.e.*, AR in QAV is more common than aortic stenosis^[^^4^^]^, even though its primary incompetency may develop into subsequent stenosis at a later stage^[^^1^^]^. Tutarel & Westhoff-Bleck^[^^13^^]^ reported that the functional status of QAV was regurgitant in 74.7%, combined stenosis and regurgitation in 8.4%, stenotic in 0.7%, and normally functioning in 16.2%. Yotsumoto et al.^[^^14^^]^ reported that, among 616 patients for an aortic valve operation, 9 (1.46%) patients had a QAV, all of whom had significant AR except one with combined aortic stenosis and mild AR. They also found 55.6% (5/9) of the AR patients had a cusp fenestration. Janssens et al.^[^^15^^]^ reported that AR was present in 56% (39/70) of the patients with a QAV. Tsang et al.^[^^2^^]^ described that 23% of the patients with a QAV had progression of AR during a mean follow-up of 5.5±3.7 years, and an association between morphological characteristics of QAV and severity of AR was found. Unequal shear stress may lead to leaflet fibrosis and incomplete coaptation^[^^16^^]^. Restriction and thickening of the aortic cusp, apparent restriction and cusp prolapse were also considered the most probable mechanisms of AR^[^^17^^]^. Thickened cusps with poor coaptation^[^^1^^]^, very thin and symmetrical cusps^[^^18^^]^, fibrous thickening, myxoid degeneration and severe calcification of the valve have been observed^[^^14^^]^. Progressive cusp fibrosis with subsequent failure of cusp coaptation over time has been suggested as the key mechanism in AR^[^^2^^]^. Unequal distribution of stress and incomplete coaptation of the cusps lead to the progression of AR^15^^]^.

## ASSOCIATED DISORDERS

QAV is usually an isolated anomaly, but other congenital heart defects can be present in 18-32% of the patients^[^^2^^,^^19^^]^, including coronary artery and coronary ostium anomalies, atrial septal defect^[^^20^^]^, ventricular septal defect^[^^21^^]^, patent ductus arteriosus^[^^22^^]^, tetralogy of Fallot^23^^]^, sinus of Valsalva fistula^24^^]^, subaortic fibromuscular stenosis^[^^25^^]^, mitral valve regurgitation^[^^26^^,^^27^^]^, mitral valve prolapse^[^^28^^]^, hypertrophic non-obstructive cardiomyopathy (with echocardiographic evidence of massive left ventricular hypertrophy and asymmetric septal hypertrophy)^[^^15^^]^, and transposition of the great arteries^[^^29^^]^, etc. Moreover, QAV was once found in a patient with Ehlers-Danlos syndrome^[^^19^^]^.

## CORONARY ANOMALIES

Coronary artery and coronary ostium anomalies are the most frequent associated disorders^[^^15^^]^. Saccular aneurysm of the non-coronary sinus and a single coronary ostium^[^^30^^]^, abnormal take-off of the right coronary artery with a small supernumerary coronary artery near the left ostium^[^^18^^]^ and displaced right coronary orifice^[^^31^^]^ have been reported to be associated with QAV. Malformation and displacement of coronary ostia is found in 10% of patients with a QAV^[^^32^^,^^33^^]^. However, Tsang et al.^[^^2^^]^ reported a lower prevalence of the malformation with an incidence of only 2%, while the left coronary ostium occluded by a small accessory aortic valve cusp was found^[^^2^^]^.

## AORTIC DILATION

Attaran et al.^[^^7^^]^ stated that in the patients studied the QAV was rarely associated with ascending aortic aneurysm and they once asserted that only 2 such cases reported in the literature. Nevertheless, Godefroid et al.^[^^18^^]^ and Bauer et al.^34^^]^ reported earlier three cases of aortic root dilation altogether. Moreover, a recent report on dysfunctional QAV surgery suggested 42% (13/31) patients had an ascending aortic diameter of ≥ 4 cm, and 7 (53.8%) patients of whom were performed concomitant repair of ascending aorta. Tsang et al.^[^^2^^]^ observed that aortic dilation was present in 29% (14/48) patients, including aortic root dilation in 36% (5/14), tubular ascending aorta dilation in 36% (5/14), and both aortic root and tubular ascending aorta dilation in 29% (4/14). Of these aortic dilation cases, 79% (11/14) were mild and 21% (3/14) were moderate. The mechanism of aortic root dilation in QAV was considered a result of elastic disruption of the aortic ring^[^^18^^]^.

## INFECTIVE ENDOCARDITIS

Infective endocarditis was found in 1.4% of the cases^[^^6^^]^. A small supernumerary cusp can be a predictive risk factor of infective endocarditis^[^^35^^]^. In patients with four equally sized cusps the risk of infective endocarditis is lower because of the lack of asymmetry or flow disturbance. In valves with unequal cusps, uneven distribution of stress and incomplete juxtaposition during diastole may lead to progressive aortic insufficiency and gradual deterioration over the years, and thus increasing the risk for endocarditis^[^^5^^]^. However, a 75-year-old man with a type A QAV with four equal-sized cusps was once reported to be affected by infective endocarditis^[^^36^^]^. The identification of a QAV with AR is important as for the high risk of endocarditis^[^^37^^]^. Takeda et al.^[^^35^^]^ reported a case of type F QAV with AR and infective endocarditis that warranted a valve replacement with a Medtronic Freestyle bioprosthesis. Pirundini et al.^[^^4^^]^ reported that one of their three patients with a QAV had recurrent endocarditis and severe AR and underwent aortic valve replacement with a bioprosthesis. Debates remained concerning the prophylaxis of infective endocarditis in patients with a QAV. Some authors advised unconditional antibiotic prophylaxis^[^^38^^]^, others recommended prophylaxis only in patients with AR with a small supernumerary cusp other than in those with trivial or mild AR with equalsized cusps^[^^15^^,^^35^^]^. However, the American College of Cardiology/American Heart Association (ACC/AHA) 2008 update on guidelines for infective endocarditis does not recommend prophylactic antibiotic treatment for the patients without the evidence of active infection^[^^39^^]^.

## CLINICAL FEATURES

The function of the QAV is usually kept normal when the patient is at the age of <18 years, and it is worsening at >40 years^[^^6^^]^. Significant valvular disorder is often present with subsequent operation performed at the fifth to sixth decade of life^[^^10^^]^. The patients' age at diagnosis was reported to be 49 (range, 6-78) years^[^^15^^]^. Most of the authors described a slight male predominance, but Janssens et al.^[^^15^^]^ presented a larger male-tofemale ratio (62% *vs.* 38%).

Clinical manifestations of the patients with a QAV depend on the functional status of the QAV and the associated disorders. Patient can be asymptomatic until the sixth decade of life^[^^6^^]^. Palpitations^18^^]^, chest pain^[^^40^^]^, shortness of breath, fatigue and pedal edema^[^^41^^]^, and syncope^[^^15^^]^ can be present. Congestive heart failure can be the presenting symptom^[^^1^^]^. Salum et al.^[^^42^^]^ reported a 56-year-old female patient with a QAV presenting with severe heart failure, heart enlargement and progressive AR. In extreme cases, sudden cardiac death may occur^[^^15^^,^^43^^]^. A decrescendo diastolic murmur at the left sternal boarder can be audible^[^^44^^]^. In patients with severe AR with left heart failure, S3 or S4 may be auscultated^[^^44^^]^. Electrocardiogram may show incomplete or complete right bundle branch block and signs of left ventricular hypertrophy^[^^18^^]^.

## DIAGNOSIS

Echocardiography was the leading mode of detection of QAVs. In majority of the patients, the diagnosis of QAV was made by echocardiography (51%), followed by surgery (22.6%), autopsy (15.6%), and aortography (6.5%)^[^^18^^]^. In a literature review including 70 cases of QAV^[^^15^^]^, the diagnosis was made by transthoracic or transesophageal echocardiography (26/70, 31.7%), necropsy (25/70, 35.7%), surgery (15/70, 21.4%), and angiography (4/70, 5.7%). The screening value of transthoracic echocardiography and diagnostic accuracy of transesophageal echocardiography for QAVs were praised^[^^6^^]^. Two-dimensional transthoracic echocardiography became available in the 1970s, and it was not used for the diagnosis of QAV until 1984^[^^45^^,^^46^^]^. It could delineate aortic valve morphology (number of cusps, degree of thickening and vegetations) and function (coaptation, regurgitation, or stenosis), aortic root size and left ventricular size, etc.^[^^5^^,^^47^^]^. Nowadays, transesophageal echocardiography has become a preferred diagnostic tool of QAVs, for not only displaying the morphology of the QAV, but also disclosing the displaced coronary ostium^[^^12^^]^. Transesophageal echocardiography usually shows a QAV with four cusps, coaptation defect and AR^[^^48^^]^. On the short axis view of the aortic valve in diastole, the commissural lines formed by the adjacent cusps shows an "X" configuration other than the "Y" configuration of the normal tricuspid aortic valve^[^^12^^]^. Color Doppler may confirm AR with central jet due to incomplete coaptation of the cusps^[^^5^^,^^6^^]^.

Cardiac computed tomography may accurately show the status of QAV, such as the failed coaptation of the leaflets and significant AR^[^^41^^]^. Additionally, it may also clearly demonstrate the location of coronary ostia, dimensions of the aorta and the conditions of the coronary arteries^[^^49^^]^. Cardiac magnetic resonance imaging may also define the morphology of QAV, AR volume and calcification of the leaflets as well^[^^48^^,^^50^^]^.

## SURGICAL INDICATIONS

**Fig. 3:**
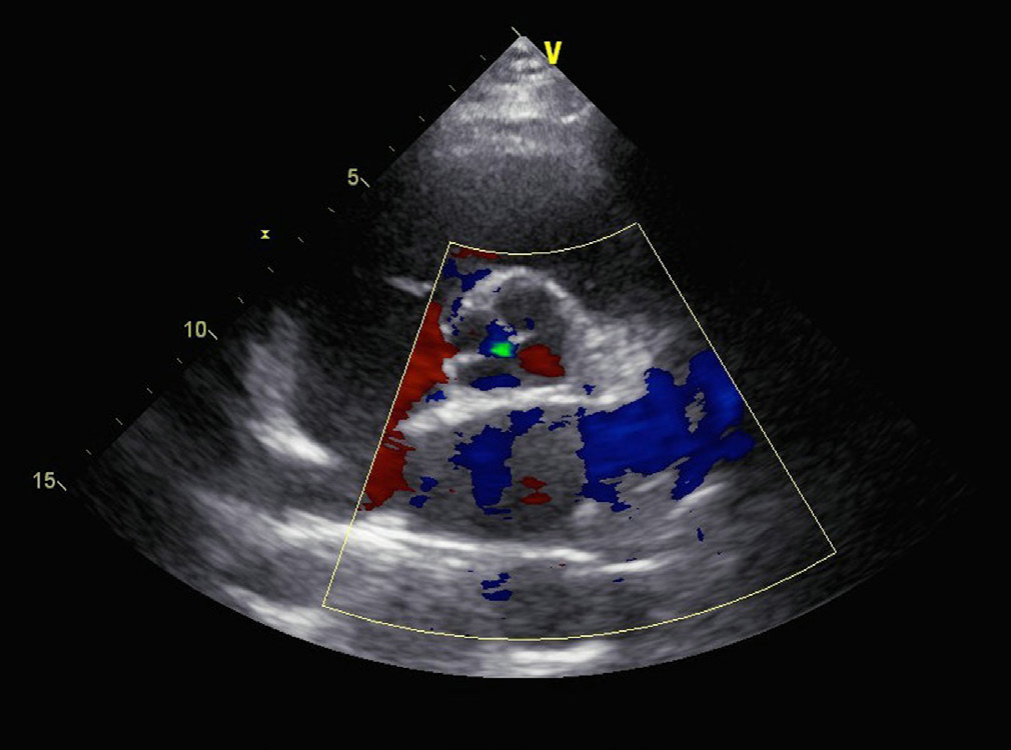
Echocardiography of a type D/type III quadricuspid aortic valve with mild aortic regurgitation.

The surgical indications for QAV are severe AR^[^^2^^]^, severe aortic stenosis^[^^51^^]^, or dysfunctional QAV associated with other lesions, such as occlusion of the left coronary ostium^[^^2^^]^. In patients with a QAV with AR, 66.7% (26/39) required an aortic valve replacement^[^^15^^]^. George et al.^[^^52^^]^ summarized previously published 15 cases of QAV and noted that only 3 (20%) required a surgical operation, in whom the surgical indications were aortic stenosis and severe AR in one, and AR associated with severe mitral valve prolapse in two patients. Tutarel^[^^53^^]^ performed simultaneously replacements of the aortic valve and root with a cryopreserved homograft for a patient with a QAV in the presence of severe calcification of the ascending aorta. We recently reported a patient with QAV (type D in Hurwitz & Roberts'^[^^8^^]^ classification and type III in Nakamura et al.'^[^^11^^]^ classification) with mild AR as identified by transthoracic echocardiography (Figure 3), who was initially referred to us due to mild exertional dyspnea, and she was advised a regular follow-up^[^^54^^]^. However, she soon went to a provincial hospital, where she received an aortic valve replacement with a St. Jude Medical mechanical prosthesis. As such, an excessive treatment was seen in both surgical indication and surgical procedure of choice.

## SURGICAL TECHNIQUES

Aortic valve replacement is not an optimal solution for these young patients, because they are exposed to valve-related risks, such as thromboembolism, prosthetic valve degeneration, infective endocarditis and bleeding, and therefore aortic valve repair could be a promising option^[^^17^^]^. The target of aortic valve repair is to restore an accurate coaptation and low transvalvular gradient with no turbulent flow and therefore to achieve a favorable long-term durability^[^^55^^]^. In addition, transcatheter aortic valve replacement is not recommended for those patients with severe AR^[^^44^^]^. Anyway, aortic valve repair started late and the choice of the procedure is usually determined on the disease severity, condition of QAV, and surgeon's preference^[^^33^^]^.

The most common repair technique is the aortic valve tricuspidalization. Iglesias et al.^[^^25^^]^ reported a case of QAV, in whom tricuspidalization by conjoining the rudimentary and right aortic valve leaflets and resection of subaortic stenosis were performed. Langer et al.^[^^56^^]^ described their QAV repair technique of neocusp creation by rudimentary commissure detachment, adjacent cusp approximation and neocusp augmentation. Schmidt et al.^[^^17^^]^ used pericardial patch augmentation and triangular resection of cusp tissue in their aortic repair technique. Kawase et al.^[^^57^^]^ introduced their technique of neocusp creation by trimming the glutaraldehyde-treated autologous pericardium. Williams et al.^[^^58^^]^ included abnormal commissure detachment, thickened tissue excision, leaflet approximation and subcommissural annuloplasty in their surgical technique of aortic valve repair. Song et al.^[^^55^^]^ presented their tricuspidization of QAV for eight consecutive patients with an at least moderate AR. Their surgical key points are pericardial leaflet reconstruction, sinotubular junction reduction and commissure coaptation suture. The latter two teams^[^^55^^,^^58^^]^ emphasized the importance of subcommissural annuloplasty and sinotubular fixation in the maintenance of the coaptation of the neocusps.

Luciani et al.^[^^59^^]^ reported their bicuspidization technique for a 68-year-old male patient with a type G QAV by joining two small non-coronary cusps to the left coronary cusp while preserving the right coronary cusp. The patient was asymptomatic at 18-month follow-up.

Additionally, Ross procedure (subcoronary technique) was reported as an alternative of treatment of QAV for decreasing the risk of aortic root dilation^[^^60^^]^. Manouguian's operation was once performed in a QAV patient with narrow annulus associated with aortic steno-insufficiency and mitral insufficiency^[^^27^^]^.

## POSTOPERATIVE COMPLICATIONS

Postoperative complications are seldom. Tsang et al.^[^^2^^]^ reported three postoperative complications, including progressive AR, transient ischemic attack and cardiac arrest in one patient each. Pirundini et al.^[^^4^^]^ reported that a patient had postoperative complete heart block, which was believed to be a result of conduction system impairment by manipulation of the supernumerary cusp of QAV that was most commonly located between the right and non-coronary coronary cusps. The overall survival rates of QAV patients were 89.9% and 84.9% at 5- and 10-year follow-up, respectively^[^^2^^]^.

## PROGNOSIS

**Fig. 4:**
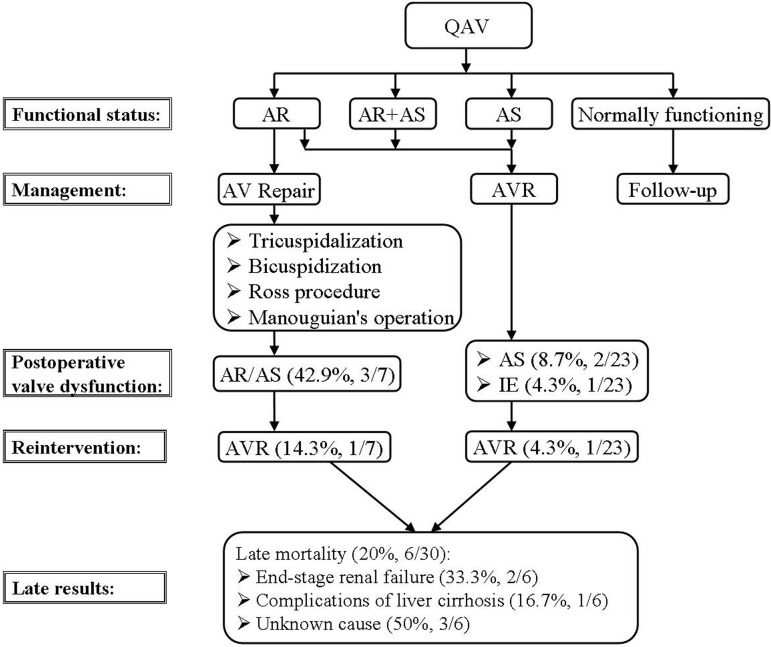
Management and prognosis of patients with a quadricuspid aortic valve^[^^33^^]^. AR=aortic regurgitation; AS=aortic stenosis; AV=aortic valve; AVR=aortic valve replacement; IE=infective endocarditis; QAV=quadricuspid aortic valve

The non-tricuspid aortic valves are less amenable to repair and durability of repair was almost uncertain as there are limited cases and scanty of reports concerning the long-term outcomes^[^^17^^]^. In the early years, Yotsumoto et al.^[^^14^^]^ reported one patient with a QAV failed for aortic valve repair and was converted to valve replacement. Song et al.^[^^55^^]^ reported eight patients with QAV with significant AR in each patient. Tricuspidization with new aortic valve leaflet created with bovine pericardium resulted in significantly improved hemodynamics in all patients and showed satisfactory early and mid-term results with no reoperative requirements. Idrees et al.^[^^33^^]^ reported the long-term outcomes of QAV patients undergoing aortic valve repair and aortic valve replacement. Three (42.9%, 3/7) patients with aortic valve repair developed regurgitation and (or) stenosis of the aortic valve and one of the three required reoperation for aortic valve replacement at 13 years after the initial operation. In comparison, 2 (8.7%, 2/23) patients developed aortic stenosis after aortic valve replacement, but without the need of re-replacement of the aortic valve. One patient of the aortic valve replacement group developed infective endocarditis and warranted re-replacement of the aortic valve. Figure 4 shows the management protocols and late results of QAV patients undergoing surgical operations.

## CONCLUSION

QAV is a rare congenital heart disease. Most of the patients with a QAV develop aortic valve incompetency at the fifth to sixth decade of life. About one-fifth of them require a surgical operation. Although tricuspidalization is a preferred repair technique for QAV with significant AR, the associated aortopathy could be a predictive risk factor of late failure of aortic repair. As not all the patients with a QAV necessarily warrant a surgical operation, decision-making in patient selection and surgical procedure of choice are crucial. The aortic valve repair of panegyric was started later and the procedural choice was determined by the feasibility concerning the QAV condition and surgeon's preference. Antibiotic prophylaxis against infective endocarditis is necessary in the QAV patients with unequal-sized cusps.

**Table:** 

**Author's roles & responsibilities**	
SMY	Study conception and design; analysis and/or interpretation of data; manuscript writing; final approval of the manuscript
